# Modeling and Characterization of Stress Relaxation Behavior of EVA–CNT Nanocomposites Under Uniaxial Loading

**DOI:** 10.3390/nano16211421

**Published:** 2026-11-04

**Authors:** Felicia Stan, Stefanut Ciochina, Ionut-Laurentiu Sandu, Catalin Fetecau

**Affiliations:** 1 Center of Excellence Polymer Processing, Dunarea de Jos University of Galati, 47 Domneasca, 800008 Galati, Romania; laurentiu.sandu@ugal.ro (I.-L.S.); catalin.fetecau@ugal.ro (C.F.); 2 Faculty of Sciences and Environment, Dunarea de Jos University of Galati, 47 Domneasca, 800008 Galati, Romania

**Keywords:** stress relaxation, Maxwell model, Prony parameters, polymer nanocomposites, carbon nanotubes, EVA

## Abstract

Understanding the time-dependent viscoelastic behavior of polymer carbon nanotube (CNT) nanocomposites is crucial for predicting their performance, particularly in load-bearing applications where the nanocomposites can be exposed to either short- or long-time loadings. In this study, the stress relaxation behavior of ethylene-vinyl acetate (EVA)–CNT nanocomposites containing 1, 3, and 5 wt% CNTs was experimentally investigated under load- and strain-controlled uniaxial relaxation conditions and modeled using the generalized Maxwell linear viscoelastic model. The results indicate that in the linear viscoelastic domain, the EVA–CNT nanocomposites retain at least 70–80% of their instantaneous modulus, and the equilibrium modulus almost doubled with increasing nanotube content from 1 to 5 wt%, yielding a 1.70- to 1.85-fold increase depending on the initial strain and relaxation period. When the strains exceed the viscoelastic limit (10–25%), all nanocomposites relaxed to about 65–70% of the initial stress. The generalized Maxwell model accurately captured the relaxation behavior of EVA–CNT nanocomposites within the linear viscoelastic domain, providing a robust fit for experimental relaxation data with relative errors not exceeding 5%. This study expands the potential application of EVA–CNT nanocomposites in engineering by providing insights into their stress relaxation behavior under both small and large deformations while highlighting areas for future improvement.

## 1. Introduction

Polymer carbon nanotube (CNT) nanocomposites are advanced materials obtained by dispersing CNTs into a polymer matrix. The unique structural, electrical, and mechanical properties of CNTs make polymer CNT nanocomposites a promising candidate for a broad range of applications [1,2,3,4,5,6,7,8]. When CNTs are added to a polymer, depending on the selection of the polymer matrix and CNT content, in addition to the enhanced mechanical performance [2,4,5,8], the resulting nanocomposites can also gain electrical conductivity [4,8,9], better thermal stability [4,6,9], and electromagnetic shielding capability [1,10]. Therefore, research and development efforts are being made to engineer polymer CNT nanocomposites with properties that meet the specific demands of specific applications, such as aerospace (lightweight, lighting-strike resistance) [4,7,8], automotive (high-strength, weight-reduction) [4,8], electronics (flexibility, piezo-resistivity, electromagnetic interface shielding) [1,10], and biomedical areas (large surface area, functionalization, flexibility and durability, conductivity) [3,5,7].

In the case of structural nanocomposite parts used in engineering applications, the main goal is the improvement of mechanical performance while preserving the processability of the polymer matrix [4,8]. However, most studies dealing with the mechanical behavior of polymer CNT nanocomposites are limited to time-independent behavior, although, in practice, polymer CNT nanocomposite components may exhibit time-dependent (viscoelastic) deformations such as stress relaxation [11,12,13,14,15,16,17,18,19,20,21,22] and creep [11,23,24,25,26].

In polymer-based composites, the time-dependent viscoelastic behavior is affected not only by the polymer matrix but also by filler content, filler dispersion, and agglomeration, with the addition of fillers generally slowing down the relaxation process [13,14,19,27,28]. Depending on the nature of the polymer matrix and filler content, several mechanisms can occur during the stress relaxation process, such as the breakage or disruption of the filler–filler and filler–polymer networks, the disentanglement of polymer-polymer networks, or the pullout of fillers from the polymer matrix [5,13,14,26,28,29].

In general, during the short-term relaxation of polymer CNT nanocomposites, the dominant mechanism is polymer segmentation motion, while the medium-term relaxation process is governed by interfacial relaxation. The polymer chains that are absorbed directly onto the CNT surfaces (bound layers) have restricted mobility, dictating the energy dissipation and viscoelastic transitions at intermediate time scales [24,29]. However, the long-term relaxation process is based on the rearrangement of the CNT network [16,26].

At small CNT content (well below the percolation threshold), CNTs act as physical obstacles, and the polymer chain motion becomes partially restricted; therefore, the relaxation rate decreases. When CNT content approaches the network threshold, the stress transfer efficiency increases since the nanotubes form interconnected structures that act as restricting sites; therefore, CNT–polymer interfacial interactions and chain slippage become more difficult. At higher CNT loadings, the relaxation process becomes much slower, and the nanocomposite behaves more like a solid (long-term residual stress increases) [13,16].

One promising application of polymer CNT nanocomposites is in electronics. In recent years, significant progress has been achieved in the development of polymer CNT nanocomposites with enhanced sensing properties, making them suitable to be used in monitoring devices for a wide range of applications, particularly for flexible strain sensors [14,30,31,32,33,34,35]. The strain sensing capacity of polymer CNT nanocomposites is governed by the change in electrical resistance relative to the applied strain [14,30,31,32,33,34,35]. Therefore, during strain sensing, the polymer CNT nanocomposites are subjected to external stimuli (small and large deformations), which create the potential for stress relaxation [14,15,19,30,31,32,33,34,35].

Ethylene-vinyl acetate (EVA) and CNTs are generally combined to create flexible, lightweight nanocomposites with improved properties [36,37,38,39,40,41,42,43,44,45,46,47,48,49], which are used in engineering applications such as flexible electronics, strain sensors, smart textiles, and soft robotics [37,40,41,42,43,44,45,46,47]. For these applications, in addition to sensitivity, which is a prerequisite, understanding stress relaxation behavior under different loading conditions is essential to avoid failures during service. However, most studies on EVA–CNT nanocomposites focus on the mechanical properties under quasi-static [36,38,39,42,49] or piezoresistive loading conditions [36,37,38,39,40,41,42,43,44,45,46,47], while the research on the time-dependent viscoelastic behavior is still insufficient. Therefore, this study investigates the stress relaxation behavior of EVA–CNT nanocomposites with 1, 3, and 5 wt% CNTs. To assess and establish the difference between the three formulations, uniaxial tensile stress relaxation tests were conducted in a wide range of applied loads and strains. The mechanisms involved in the relaxation process were analyzed using the generalized Maxwell linear viscoelastic model.

## 2. Experimental

### 2.1. Material and Sample Preparation

The nanocomposites considered in this study consist of the ethylene-vinyl acetate (EVA) copolymer (Alcudia PA-420, Repsol [50]) with 20% vinyl acetate, reinforced with 1, 3, and 5 wt% thin-multi walled carbon nanotubes (CNT, NC7000™, Nanocyl S.A., Sambreville, Belgium) [51]. These CNT concentrations were selected to ensure good processability while preserving the mechanical properties of EVA matrix, particularly the ductility. The EVA–CNT nanocomposites, supplied by Nanocyl S.A. (Belgium), were prepared by melt mixing. According to the manufacturer, the nanotubes were directly incorporated into the molten polymer (140 °C) via a side feeder. The test specimens (Type I according to ISO-527 [38,52]) were manufactured by injection molding using an Arburg injection molding machine (Allrounder 320, Arburg, Lossburg, Germany). The injection molding parameters were set as follows: 140 °C melt temperature, 30 °C mold temperature, 70 MPa injection pressure, 15 cm^3^/s injection speed, 10 s holding time, and 20 s cooling time.

### 2.2. Stress Relaxation Tests

For the current study, to investigate the time-dependent relaxation behavior of the EVA–CNT nanocomposites, load- and strain-controlled uniaxial relaxation tests were performed on a universal testing machine (M350-AT, Testometric Co. Ltd., Rochdale, UK) with a displacement reading resolution of 0.001 mm with 0.025 N accuracy. The stress relaxation tests were performed at room temperature with a crosshead speed of 50 mm/min, and three fresh specimens were used for each relaxation test. Before stress relaxation testing, uniaxial tensile tests were conducted at a crosshead speed of 50 mm/min to evaluate the mechanical properties (e.g., elastic modulus and yield point) and estimate the upper limit of the linear viscoelastic region (LVR). The stress–strain curves were determined directly from the load-crosshead displacement curves.

In the load-controlled relaxation tests, the specimens were stretched to the same initial load level (50 and 100 N, respectively, to ensure that the stress measurements remain within the elastic zone or near the yield point). After reaching the predefined load, the crosshead was held constant at the corresponding strain level, and the force was monitored for a time span of 12 h.

In the strain-controlled relaxation tests, i.e., a linear ramp to a prescribed strain, the specimens were stretched to a predefined strain (1, 10, and 25%) with the same crosshead displacement of 50 mm/min. Following the ramp loading to the defined strain level, the crosshead was stopped, and the specimen was held over a time span of 1 h while the force was monitored. It should be noted that the initial strains of 10% and 25% were selected to investigate the stress relaxation behavior under large strains since—in many engineering applications—the relaxation response may occur outside the LVR.

## 3. Modeling of Stress Relaxation Behavior

### 3.1. Generalized Maxwell Model

The constitutive model used to describe the viscoelastic behavior of EVA–CNT nanocomposites is based on the generalized Maxwell model that consists of a spring of elastic modulus *E*_∞_ in parallel with Maxwell elements [18,53,54,55], as shown in Figure 1.

Since the elements of the generalize Maxwell model are connected in parallel, the strain *ε*(*t*) is identical across all elements (branches), while the total stress *σ*(*t*) splits among them. Thus, the total relaxation stress can be expressed as follows [53,54]:

(1) $$\sigma \left(t\right)=\sigma_{\infty }+\sum_{k=1}^{N}\sigma_{k}\exp\left(-\frac{t}{\tau_{k}}\right)$$

where *σ*_∞_ is the long-term equilibrium stress, *σ_k_* is the stress contribution of the *k*-th Maxwell element, and *N* is the number of Maxwell elements.

In linear viscoelasticity, for uniaxial stress relaxation under applied constant strain, the time-dependent behavior can be described by the relaxation modulus given by the following equation [53,54]:

(2) $$E\left(t\right)=\frac{\sigma \left(t\right)}{\varepsilon_{0}}$$

where *ε*_0_ is the initial constant strain (step strain).

The relaxation modulus is generally given in the form of Prony series [53,56,57].

(3) $$E\left(t\right)=E_{\infty }+\sum_{k=1}^{N}E_{k}\exp\left(-\frac{t}{\tau_{k}}\right)$$

where *E*_∞_ is the-long term equilibrium relaxation modulus (as *t* → ∞), *τ_k_* = *η_k_*/*E_k_* is the *k*-th relaxation time (relaxation time spectrum), and *E_k_* is the *k*-th Prony coefficient (relaxation modulus, spring/stiffness constants of the *k*-th Maxwell element). The values *τ_k_* and *E_k_* form a discrete relaxation spectrum.

The instantaneous modulus, or the glassy modulus (as *t* = 0), is given by the following equation [54]:

(4) $$E_{0}=E_{\infty }+\sum_{k=1}^{N}E_{k}$$

In the case of polymer CNT nanocomposites, each term in the Prony series represents a mechanism occurring at a specific time scale, such as chain (segmental) motion, polymer chain disentanglement, CNT network rearrangement including CNT–CNT and polymer–CNT breakage, or interfacial slippage and debonding [11,13,16,23]. Segmental motion and chain disentanglement are two mechanisms specific to the polymer matrix. Since CNTs introduce a large interfacial area where polymer chains are constrained [24,29], polymer chains near the CNT surface have restricted mobility, i.e., the CNT network creates delayed relaxation behavior. On the other hand, the load is transferred from the polymer matrix to CNTs and back to the polymer; thus, interfacial slipping or debonding can occur over time [16,23].

Small *τ* values (e.g., fast relaxation process, short-term relaxation) describe the segmental motion of polymer chains; while the response of the polymer matrix dominates the relaxation behavior, the CNTs have a minimal contribution. Medium *τ* values (e.g., intermediate-term relaxation) describe interfacial effects or disentanglement. In this case, the contribution of the CNTs to the relaxation process increases. An interphase zone forms around the nanotubes where polymer chains are tightly adsorbed or pinned to the CNT walls [24,29]. This chain immobilization delays the relaxation process, causing the nanocomposite to hold onto stress longer than the neat polymer. Long-term relaxation (larger *τ* values) is generally characterized by two mechanisms: interfacial slipping and CNT network rearrangement. In this case, the effect of CNTs is dominant. Under continuous uniaxial tension, the polymer matrix relaxes intensively, transferring the load to the CNT network. If the strain is high enough, interfacial sliding occurs at the polymer–CNT interface due to weak van der Waals forces. Simultaneously, the percolated CNT network undergoes structural rearrangement, slowly breaking and reforming contact points to release energy [11,13,16].

The values of *τ_k_* and *E_k_* can be estimated by minimizing the least-squares error to fit a set of experimental data with Equation (3) for 2*N* unknown parameters.

The initial guess for the relaxation times *τ_k_* in Equation (3) can be estimated using the approach proposed by Ref. [58]:

*τ_k_* = *τ*_1_ ⋅ 10^(*k*−1)⋅Δ^ (5)

where

(6) $$\Delta =\log\left(\frac{\tau_{\max}}{\tau_{\min}}\right)\cdot \frac{1}{N-1}$$

in which *τ*_min_ and *τ*_max_ are the first and last available data durations of the relaxation experiment.

As the number of the Maxwell elements increases, approaching infinity (*N* → ∞), the relaxation function (3) can be expressed in integral form [56,59]:

(7) $$E\left(t\right)=E_{\infty }+\left(E_{0}-E_{\infty }\right)\cdot \int_{0}^{\infty }h\left(\tau \right)\cdot \exp\left(-\frac{t}{\tau }\right)\mathrm{d\tau}$$

where *h*(*τ*) represents the continuous relaxation time spectrum.

For convenience in the computation, the relaxation function (7) can be normalized by the difference *E*_0_ − *E*_∞_, leading to [56,59]:

(8) $$R\left(t\right)=\frac{E\left(t\right)-E_{\infty }}{E_{0}-E_{\infty }}=\int_{0}^{\infty }h\left(\tau \right)\cdot \exp\left(-\frac{t}{\tau }\right)\mathrm{d\tau}$$

The integral (8) can be solved by utilizing an inverse Laplace transform.

The long-term relaxation ratio is defined as:

(9) $$R_{\infty }=\frac{\sigma_{0}-\sigma_{\infty }}{\sigma_{0}}\cdot 100\%$$

It can be computed to describe the experimental relaxation behavior at time *t* = ∞.

### 3.2. Prony Parameter Fitting

The parameters of the generalized Maxwell model, used to study the viscoelastic behavior, are calculated in MATLAB (R2025a) using the nonlinear least-squares method [60]. The mathematical algorithm consists of minimizing the sum of squares of the residuals between the experimental data and those obtained by the developed model [54,60]

(10) $$C=\frac{\sum_{i=1}^{M}{\left(E_{i}^{\exp}-E_{i}^{\mathrm{fit}}\right)}^{2}}{\sum_{i=1}^{M}{\left(E_{i}^{\exp}\right)}^{2}}$$

where $E_{i}^{\exp}$ and $E_{i}^{\mathrm{fit}}$ denote the experimental and predicted modulus, and *M* is the number of time points.

The residuals were calculated in logarithmic space, providing a more balanced weighting across the time domain. Additionally, a series of constraints were imposed on the model optimization. The first condition refers to the positivity of the equilibrium modulus and the elastic moduli, and the second restriction is given by the fact that the relaxation times must be within the time interval used in the experimental part. The objective function minimization uses the Trust Region Reflective algorithm [60], in accordance with the restrictions imposed.

The reliability and accuracy of the model were evaluated by calculating the coefficient of determination (*R*^2^) and the sum of squares (*SSq*) of the relative error. The *SSq* was used as an overall measure of the error of the predicted model.

The range of relaxation times for the fitting process should be the same as the range of measured data, or about a decade or two larger than the complete range of experimental data, as shown by Ref. [61]. To model the viscoelastic behavior with Prony series, at least one relaxation time per decade—equally spaced along the logarithmic time axis—should be taken. Moreover, it was shown that two relaxation times per decade yield some improvement, while considering more than two relaxation times per decade will not bring any substantial improvement into the quality of fitting [59,61,62].

## 4. Results and Discussion

### 4.1. Morphology of EVA–CNT Nanocomposites

Figure 2 displays the SEM images of the cryo-fractured samples of EVA nanocomposites with 1, 3, and 5 wt% CNTs. Depending on the CNT content, an analysis of SEM images highlights three main characteristics in the EVA–CNT nanocomposite morphology: isolated CNTs, CNT bundles, and CNT agglomerates.

At the lower content (1 wt%), CNTs (bright spots) are dispersed randomly in the form of isolated (single) nanotubes and small bundles (Figure 2a). Also, it is observed that CNTs are wrapped by a large amount of the EVA matrix and primarily oriented along the flow direction (perpendicular to the cross-section). At 3 wt%, in addition to isolated CNTs and CNT bundles, small CNT agglomerates are visible (Figure 2b). At 5 wt%, the number of CNT agglomerates increased, and between these agglomerates, loosely connected CNTs and even isolated CNTs are observed (Figure 2c).

Comparing the SEM images of nanocomposite samples with different CNT contents, no major differences in the size of CNT agglomerates were observed, which are roughly 1–3 µm in diameter; this could be attributed to high shear forces developed during the injection molding process [38,39], which break down the bigger CNT agglomerates that may exist in pellets and could not be dispersed during the melt mixing process. These results are similar with other results reported in the literature [14,23,26,36,63].

### 4.2. Relaxation Behavior of EVA–CNT Nanocomposites

#### 4.2.1. Classification of Relaxation Behavior

Figure 3 shows representative nominal stress–strain curves for the EVA–CNT nanocomposite as a function of the CNT content. All nanocomposites exhibit a bi-linear stress–strain relationship without a pronounced yield point. The overall load-bearing capacity increases with increasing CNT content, accompanied by an increase in linear elastic zone (i.e., proportional limit) and elastic limit (i.e., the point where material begins to experience nonlinear deformation), as well as a decrease in yielding (i.e., 2% offset deformation) and ductility (i.e., strain at break). An evaluation of the elastic moulus, i.e., the slope of the stress–strain curve in the elastic region, yielded values of 54, 72, and 92 MPa for 1, 3 and 5 wt% CNT, respectively.

Based on the experimental data in Figure 3, two relaxation regions were identified depending on whether the nanocomposite response is (i) in the linear viscoelastic region (LVR, below the yield point) or (ii) in the non-linear viscoelastic region (non-LVR, above the yield point).

Since the EVA–CNT nanocomposites do not present a sharp, distinct yield point, the upper limit of the LVR was defined as the 2% offset yield strain, which reached 9.8, 8.2, and 6.9% for 1, 3, and 5 wt% CNTs, respectively. Hence, for the load-controlled tests, the response is linear viscoelastic (corresponding to initial strains up to 6%), whereas, for the strain-controlled tests, the response is linear viscoelastic at 1% strain and becomes nonlinear viscoelastic at 10% and 25% strain.

#### 4.2.2. Load-Controlled Stress Relaxation Behavior of EVA–CNT Nanocomposites

Figure 4 shows the typical stress relaxation curves for the EVA–CNT nanocomposite as a function of CNT content for the two initial loads of 50 N and 100 N. Zero time was taken as the time when the crosshead was stopped—the maximum prescribed load was reached. The relaxation stress was calculated under the assumption that no changes in the cross-section area occur during the relaxation test. The parameters related to the relaxation of EVA–CNT nanocomposites (e.g., the initial stress, *σ*_0_, initial strain, *ε*_0_, equilibrium stress, *σ*_∞_, and relaxation ratio, *R*_∞_) are presented in Table 1.

As illustrated in Figure 4, the relaxation of EVA–CNT nanocomposites occurs gradually, reaching a steady state after about 1 h of relaxation and decreased by only 15% and 10% for initial loads of 50 N and 100 N, respectively, after being held for 12 h. Moreover, the EVA–CNT nanocomposites relaxed to different stress levels depending on the initial loading (initial strain), and none of them relaxed completely. It is also observed that the initial relaxation strain decreases with increasing CNT content as the material becomes more rigid (Table 1).

To further analyze the effect of CNTs on stress relaxation, the normalized stress obtained for the first 100 s was plotted in Figure 5. Stress relaxation is likely governed by fast, local segmental motion of the polymer chains and CNTs do not locally influence the short-range relaxation. Consequently, the normalized stress curves overlap. As the relaxation time increases, the polymer chains interact with CNTs, and the curves start to separate.

As can be seen in Table 1, the nanocomposite with 1 and 3 wt% CNTs retains a slightly higher percentage of its initial stress as compared with 5 wt% CNTs. On the other hand, increasing CNT content from 1 to 5 wt% increases the relaxation ratio after 12 h, indicating that the nanocomposite with 5 wt% relaxes faster than the nanocomposite with 1 and 3 wt% CNTs, meaning that the material dissipates more internal stress over time when held at a constant strain.

The stress relaxation behavior is hypothesized to be governed by several competing mechanisms that, generally, are activated depending on the applied load/strain and CNT content. At low CNT content (e.g., 1 wt%), the polymer matrix dominates the relaxation process as CNTs are likely isolated or form weak, sparse pathways (Figure 2a). As the content increases to 3 wt%, CNTs form a percolation network, while higher CNT content (e.g., 5 wt%) creates a denser, percolating network (Figure 2c). Under constant strain during a 12 h relaxation period, this dense network may potentially undergo physical structural rearrangement—such as inter-tube or polymer–CNT interfacial sliding, CNT–CNT junction breakage, or network reorganization, as documented in the literature [11,12,13,23,26,64,65]. This provides an additional pathway for stress dissipation that does not exist at lower CNT contents, leading to lower equilibrium stress (higher relaxation at equilibrium, Table 1). On the other hand, due to the high aspect ratio, at higher CNT content (5 wt%), the total surface area of the nanotubes interacting with the polymer matrix increases; when the nanocomposite is held at constant strain, the polymer chain segments experience localized shear forces against the CNT surfaces. The energy is dissipated through interfacial friction and micro-slippage between the polymer matrix and nanotubes, increasing the relaxation ratio [11,13,24,29]. Moreover, higher filler contents may increase the mobility of the amorphous phase due to CNT acting as restricted sites [65], or it may introduce microscopic defects and void [25,66,67], allowing the polymer matrix surrounding the nanotubes to relax more rapidly.

When the load doubles from 50 to 100 N (e.g., the initial stress doubles from 1.25 to 2.5 MPa), the nanocomposites show better long-term performance as they retain a higher percent of the initial stress and relax slower after 12 h (e.g., the relaxation ratio decreases, Table 1), but the tendency with CNTs remains the same. Doubling the initial load causes a sharp increase in the initial elastic stress response, while the viscous component of the material has a physical limit of how fast it can dissipate that energy. On the other hand, when the initial load is doubled, the polymer chains are stretched out. The localized stress concentrations lock the neighboring polymer chains tightly against the rigid CNT walls [24,29]. Under continuous loading, these trapped polymer segments are immobilized, preventing rearrangement or sliding, which restricts viscoelastic relaxation [21,24,29].

Statistical analysis indicates a highly significant effect of the initial relaxation load on the long-term relaxation stress (*p*-value = 0.0001), whereas the influence of CNT content is marginal (*p*-value = 0.06) at the 5% significance level.

To obtain the relaxation parameters that can be used for further predictions, the experimental relaxation data in Figure 4 were modeled using the generalized Maxwell model, as shown in Equation (3), by minimizing the least-squares coefficient (Equation (10)). Although the ramp loading is not an ideal ramp, the experimental ramp time was about 15 s, the effect of ramp history was neglected [57,61,68,69]. According to Ref. [68], the ramp data can be ignored if the experimental relaxation time is more than 100 times larger than the ramp time. In this case, the relaxation modulus, obtained from the relaxation response after the ramp, is a good approximation of the true relaxation modulus.

The experimental data cover about 4.5 decades; thus, in this analysis, it was determined that five Prony terms give a good fitting to the stress relaxation data. During the fitting procedure, the values of the relaxation times were fixed at equal intervals along the logarithmic time scale, and only the associated coefficients were determined by minimizing Equation (10). It should be noted that the initial guesses for *τ_k_* were estimated based on Equations (5) and (6), and then refined to 1 s, 10 s, 10^2^ s, 10^3^ s, and 10^4^ s.

The Prony parameters obtained through curve fitting (Equation (10)) for the ideal step loading—i.e., neglecting the ramp loading part—are given in Table 2 and Table 3. It should be noted that the instantaneous modulus *E*_0_ was computed with Equation (4). The experimental relaxation modulus and the numerical predictions using the Maxwell model with five elements are illustrated in Figure 6. The predicted relaxation modulus has been separated by one decade for clarity. The Maxwell model captures very well the relaxation in EVA nanocomposites (*R*^2^ ≈ 0.98 to 0.997). The maximum relative error between the experimental and predicted values falls to under 5%.

Under both loading conditions, both the instantaneous modulus *E*_0_ and equilibrium modulus *E*_∞_ of the EVA nanocomposite increase substantially as the CNT content increases from 1 to 5 wt%. In fact, these values almost doubled (Table 2 and Table 3), showing clear mechanical reinforcement. The increase in both the instantaneous modulus and long-term equilibrium modulus may indicate that CNTs formed a percolated network that restricts the free movement of flexible EVA chain segments [38,39].

The absolute drop in modulus during the relaxation process (Δ*E* = *E*_0_ − *E*_∞_) is more than double, indicating that introducing more CNTs increases the total amount of stress energy that the nanocomposite can dissipate over time. Moreover, the relative relaxed modulus (Δ*E*/*E*_0_) increases steadily with increasing CNT content. For example, at 50 N, the Δ*E*/*E*_0_ increased from about 33 to 38% as the CNT content increased from 1 to 5 wt%. On the other hand, the Δ*E*/*E*_0_ decreases when the EVA nanocomposites are subjected to higher loads; for instance, at 5 wt% CNTs, it drops from about 38% at 50 N to 32% at 100 N. At 100 N, the EVA nanocomposite is stretched towards the upper limit of the elastic zone, activating more CNTs to sustain the load. The large deformation of the matrix may induce higher interfacial shear stress across the polymer–CNT interface [13,24,65], slightly restricting molecular slippage over 12 h. Additionally, under long-term high deformations, CNTs can potentially slip, and the CNT network is prone to breaking, which reduces their reinforcing efficiency [13,24,65].

#### 4.2.3. Strain-Controlled Relaxation Behavior of EVA–CNT Nanocomposites

The representative stress relaxation curves for the EVA–CNT nanocomposites stretched to different initial tensile strains (1, 10, and 25%) are presented in Figure 7. Zero time was taken at the time when the prescribed strain value was reached, and the crosshead was stopped. Table 4 presents the corresponding stress relaxation parameters.

The initial relaxation stress *σ*_0_ and the long-term equilibrium stress *σ*_∞_ increased with increasing initial strain and nanotube wt%, as can be seen in Table 4. At each initial strain level, the *σ*_0_ value increased due to the reinforcing effect of CNTs; the higher the nanotube wt% is, the higher the initial stress. This indicates that the external loads are efficiently transferred to the nanotubes in terms of shear stress through the CNT–EVA interface. Furthermore, at each initial strain level, the nanocomposite containing 5 wt% CNTs retained a greater *σ*_∞_ value after 1 h compared to those with 1 and 3 wt% CNTs.

When the first 100 s of the experimental relaxation data were analyzed (Figure 8a), it was observed that at 1% initial strain, the normalized stress curves overlap regardless of the nanotube content. This behavior is likely due to the fact that the EVA matrix relaxes at its intrinsic rate. As the relaxation time increased, the polymer chain slipping interacts with CNTs and the curves start to separate. At higher initial strains (10% and 25%), the overlapping zone reduced substantially, indicating that the polymer matrix is stressed out and CNTs start to contribute to the relaxation process event at very short relaxation times (Figure 8b,c).

Table 4 indicates that the relaxation ratio increases with increasing initial strain, suggesting that the nanocomposites relax faster at higher initial strains. At 1% initial strain, although a higher CNT content provides superior reinforcement, the nanocomposites with 1 and 3 wt% exhibit faster relaxation compared to the 5 wt%.

At 25% initial strain, the nanocomposite with 5 wt% CNTs exhibited higher initial and equilibrium stresses after 1 h. However, the differences in the stress relaxation ratios among the three CNT contents were negligible, with all nanocomposites relaxing at approximately the same rate (33–35%). This behavior can be attributed to the fact that when the strains exceed the linear viscoelastic limit (such as past the yield strain), due to the large amount of deformation, different internal mechanisms can be activated that affect the CNT network, such as micro-cracking, molecular chain or nanotube slippage, interfacial debonding, or CNT rearrangement [13,23,25,65]. Once the CNT network is disrupted, the polymer matrix begins to govern the relaxation process; consequently, the relaxation behavior becomes matrix-dominated, with all nanocomposites showing similar relaxation ratio, regardless of the CNT content (Table 4).

At the 5% significance level, statistical analysis indicates that the initial strain has a significant effect on both the initial relaxation stress and long-term relaxation stress (*p*-value = 0.001), whereas the influence of CNT content is significant only on the long-term relaxation stress (*p*-value = 0.044).

The experimental data at 1% initial strain lie within the LVR and were fitted to the generalized Maxwell model in Equation (3). The parameters *τ_k_* (relaxation time) and *E_k_* (relaxation modulus) were estimated by minimizing the least-squares of the residuals between the experimental data and those obtained by the developed model (Equation (10)). It should be noted that the experimental data cover 3.5 decades, and thus it was determined that four relaxation Prony terms (*N* = 4 in Equation (3)) cover the stress relaxation data. The constants determined by curve fitting the generalized Maxwell viscoelastic models along with the fitting performance (*R*^2^ and *SSq*) are presented in Table 5. The tandem use of the *R*^2^ (with a value tending to 1) and the *SSq* (close to 0) indicates that the Maxwell model with four Prony terms is well suited for predicting the relaxation behavior of EVA–CNT nanocomposites in the linear viscoelastic domain. Figure 9 compares the results of the Maxwell relaxation modulus to the experimental data for 1% of the initial strain.

Normalized spectra can provide information on the fraction of the initial stiffness allocated to each relaxation mode (*g_k_* = *E_k_*/*E*_0_). Table 6 presents the normalized spectra for the EVA–CNT nanocomposites along with the equilibrium elastic fraction (*g*_∞_ = *E*_∞_/*E*_0_), computed based on the relaxation moduli in Table 5.

At 1% strain, the efficiency of the CNT network is considerable; at 5 wt% CNTs, the instantaneous modulus of the nanocomposite increased by about 64% (from 63 to 104 MPa) and the relaxed modulus increased by 73% (from 49 to 85 MPa) compared to the 1 wt% formulation. Moreover, the 5 wt% nanocomposite retains the highest percentage of its initial stiffness over a 1 h relaxation period, with about 82% remaining as a permanent elastic response (Table 6). The decrease in relaxation times across all three formulations may suggest the formation of an interconnected network through which a higher portion of the overall load is transferred, forcing a faster transition toward equilibrium.

The 3 wt% nanocomposite has the lowest equilibrium retention (76%) and the highest relative transient relaxation weights (*g*_1_ to *g*_2_), indicating that near the percolation wt%, the system is highly dynamic—yielding a higher fraction of structural relaxation.

For all three CNT contents, *g*_4_ is the largest value; this may indicate that the bulk rearrangement of polymer chains over a long relaxation period represents the primary mechanical relaxation process.

Table 6 indicates that the nanocomposite with 5 wt% possesses very good dimensional stability (reflected by a higher *g*_∞_ value) but lower damping capacity. On the other hand, the 3 wt% formulation exhibits the best impact absorption and energy dissipation behavior (reflected by higher *g*_1_ to *g*_4_ values).

While the generalized Maxwell model provides a good description of the stress relaxation behavior for EVA–CNT nanocomposites at 1% initial strain, it cannot capture their relaxation behavior at large strains (10% and 25%%). Therefore, to create an analogy with the Maxwell model, the exponential stress relaxation function (Equation (1)) with four exponential terms (*N* = 4) was used to model the experimental data at 10% and 25% strain. The fitted model parameters (*τ_k_* and *σ_k_*), along with the fitting performance (*R*^2^ and *SSq*), are presented in Table 7 and Table 8. The exponential relaxation function captures the experimental relaxation behavior of the EVA–CNT nanocomposites under large strains very well (*R*^2^ > 0.999). Consequently, these model parameters can be used to predict the nonlinear stress relaxation in engineering applications when deformations exceed the LVR.

## 5. Summary and Conclusions

In this study, the stress relaxation behavior of EVA–CNT nanocomposites with 1, 3, and 5 wt% CNTs was analyzed under small and large strains using two different experimental modes, such as load- and strain- controlled uniaxial tensile tests. The experimental stress relaxation data were fitted to the generalized Maxwell linear viscoelastic model, and the Prony relaxation parameters were calculated using the nonlinear least-squares method. The main findings from the study are as follows:

- The stress relaxation response of the three EVA–CNT nanocomposites (1, 3 and 5 wt%) remains within the LVR up to a 6% strain and exhibits a clear transition to non-linear behavior at higher strains (near or above the yield point).
- The generalized Maxwell model accurately captured the relaxation behavior of EVA–CNT nanocomposites within the LVR, providing a robust fit for experimental relaxation data with relative errors not exceeding 5%.
- Within the LVR, the EVA–CNT nanocomposites retain at least 70–80% of their instantaneous modulus. More importantly, increasing the CNT content from 1 to 5 wt% nearly doubled the equilibrium modulus, yielding a 1.70- to 1.85-fold increase depending on the initial strain and relaxation period.
- Under high strains (10% and 25%), the relaxation behavior of EVA–CNT nanocomposites became matrix-dominant, with all three formulations exhibiting a similar stress relaxation ratio of 30–35%.
- From a practical point of view, the nanocomposite with 5 wt% exhibits very good dimensional stability but lower damping capacity. Conversely, if the application requires impact absorption or energy dissipation, the nanocomposite with 3 wt% is the best candidate.

The present modeling framework assumes linear viscoelastic behavior, and the material parameters in terms of Maxwell relaxation modulus are reliable within the spectrum of strain or load levels used during the original experimental curve-fitting process. However, during a given application, the nanocomposites can be subjected to different types of loads or strains that may exceed the purely viscoelastic domain, and the generalized Maxwell model cannot be applied for capturing the relaxation response. Therefore, future work will investigate the applicability of other relaxation models, including nonlinear Maxwell and non-Maxwellian viscoelastic models, to capture stress relaxation behavior under large strains. In addition, as the properties of these nanocomposites extend across different scales, future work will address time-dependent behavior at micro- and nanoscales.

## Figures and Tables

**Figure 1:**
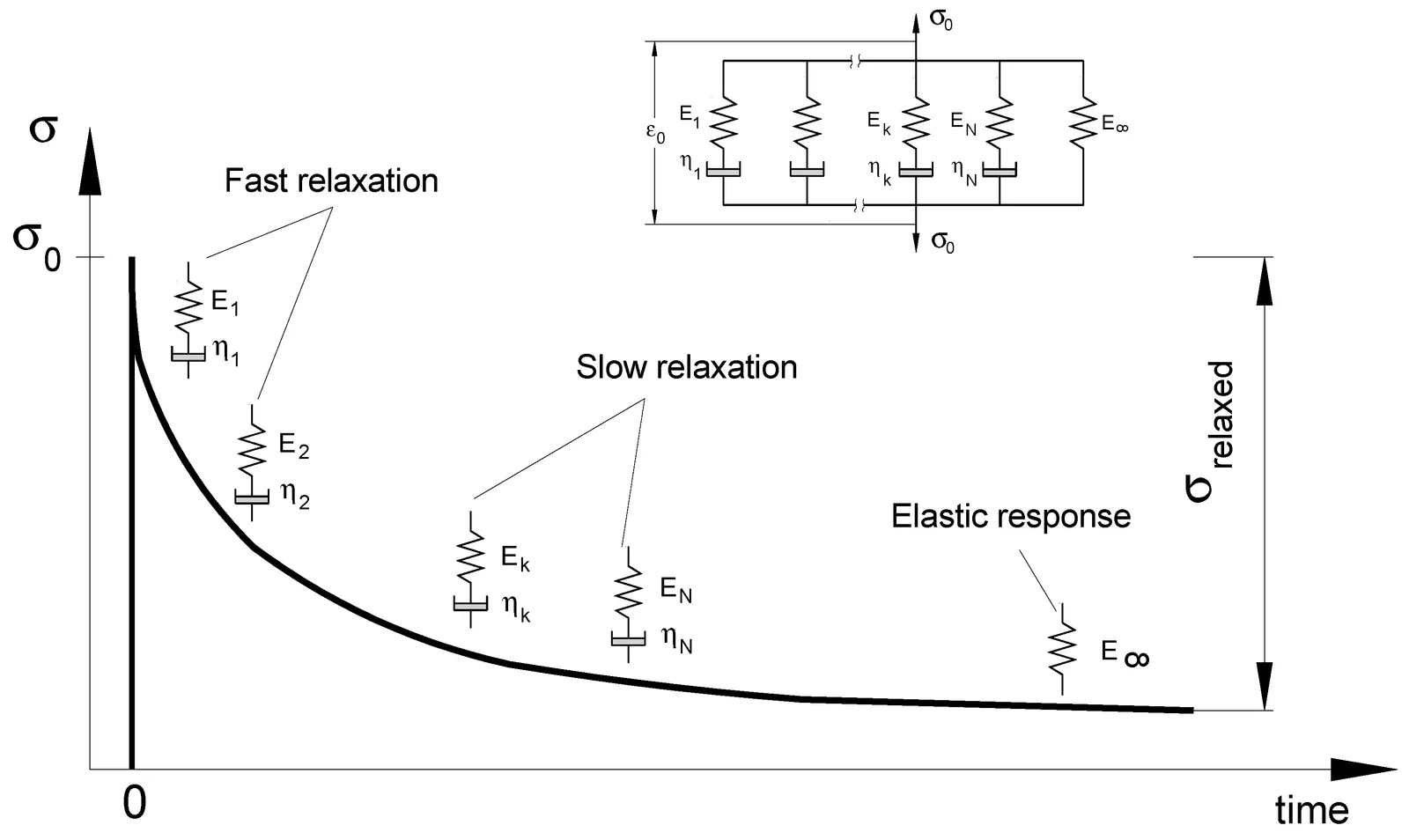
Stress relaxation curve and the generalized Maxwell model with relaxation elements connected in parallel.

**Figure 2:**
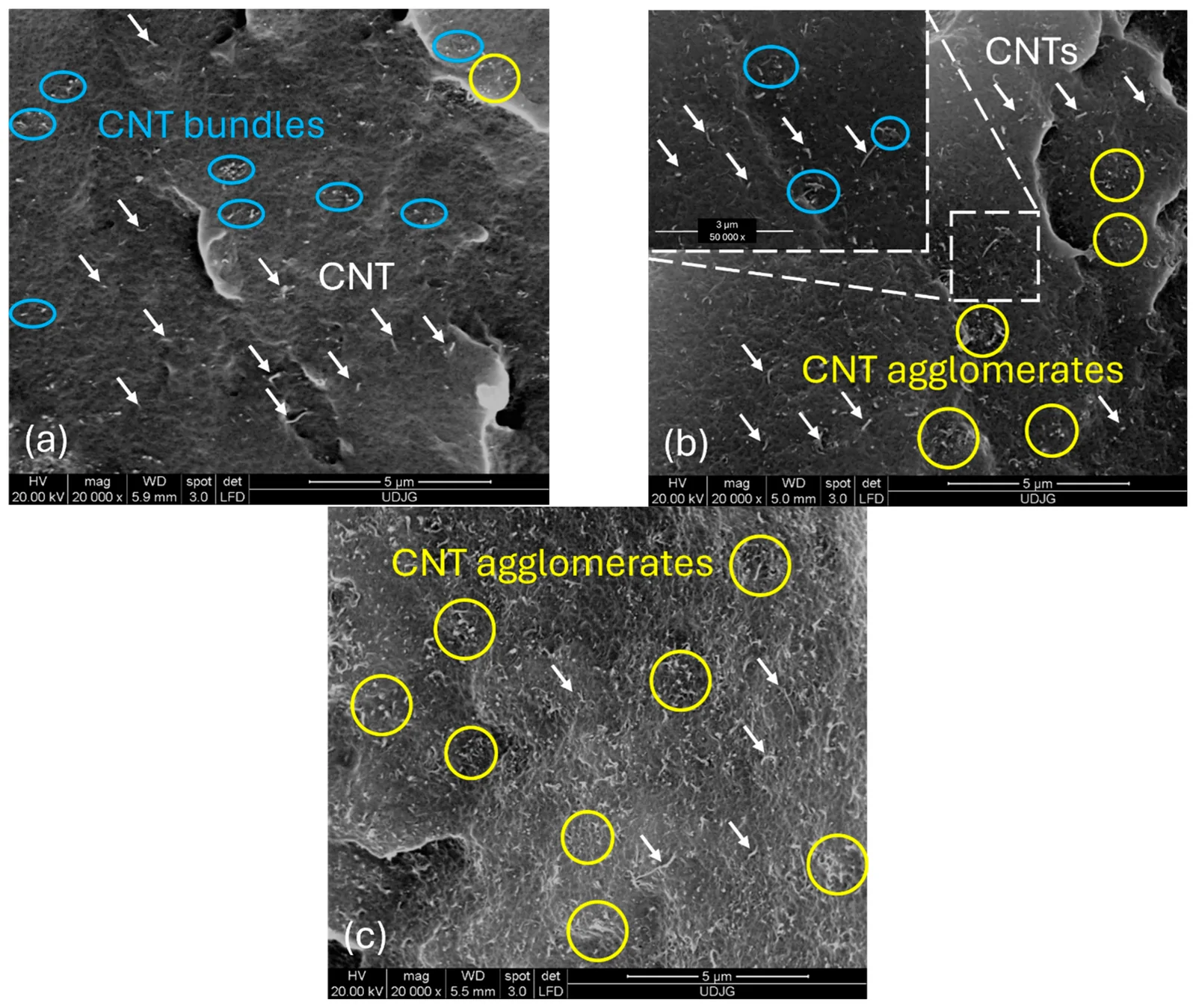
SEM image of EVA–CNT nanocomposite with (**a**) 1 wt%, (**b**) 3 wt%, and (**c**) 5 wt% CNTs.

**Figure 3:**
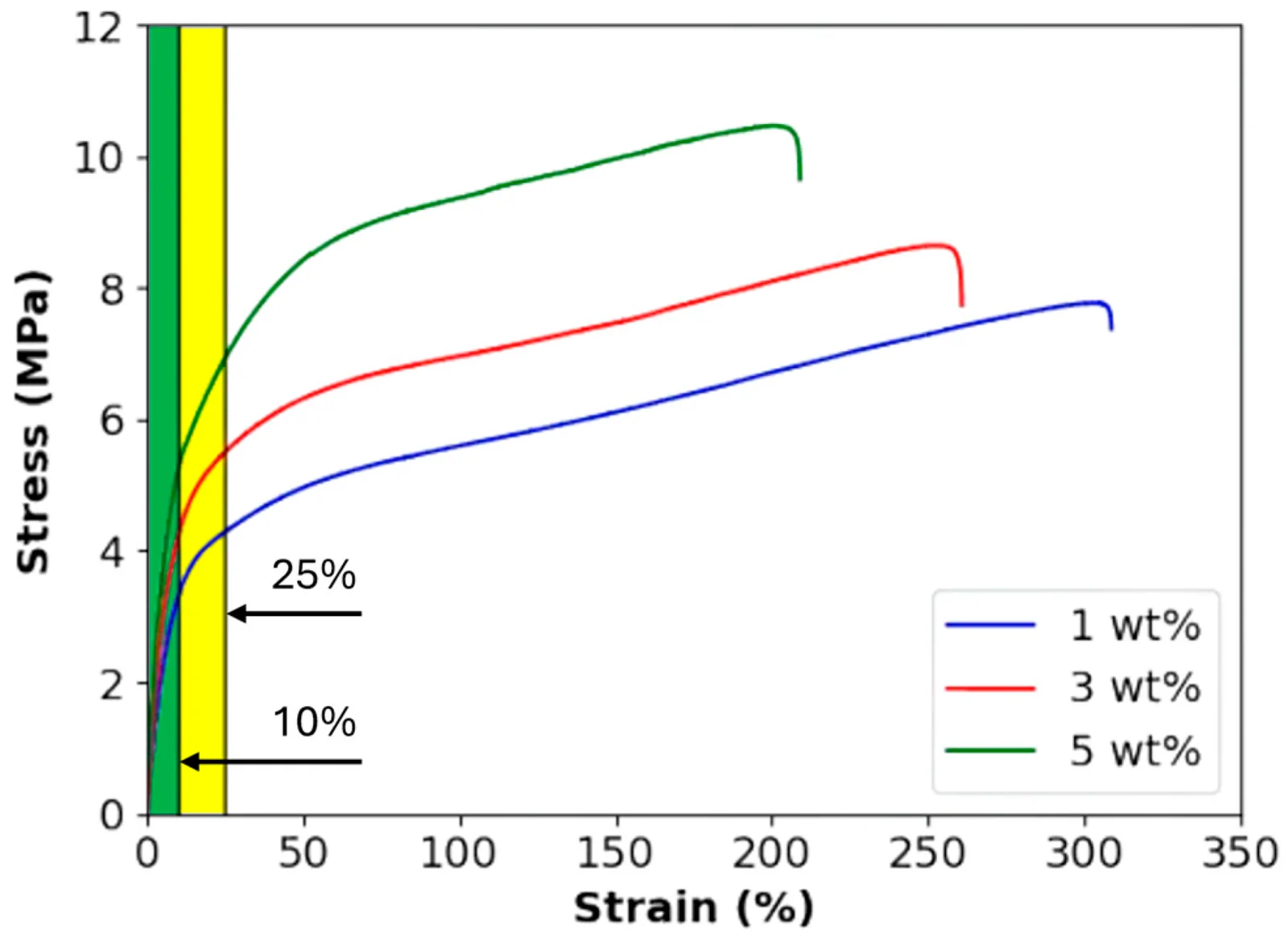
Stress–strain curves for EVA–CNT nanocomposites.

**Figure 4:**
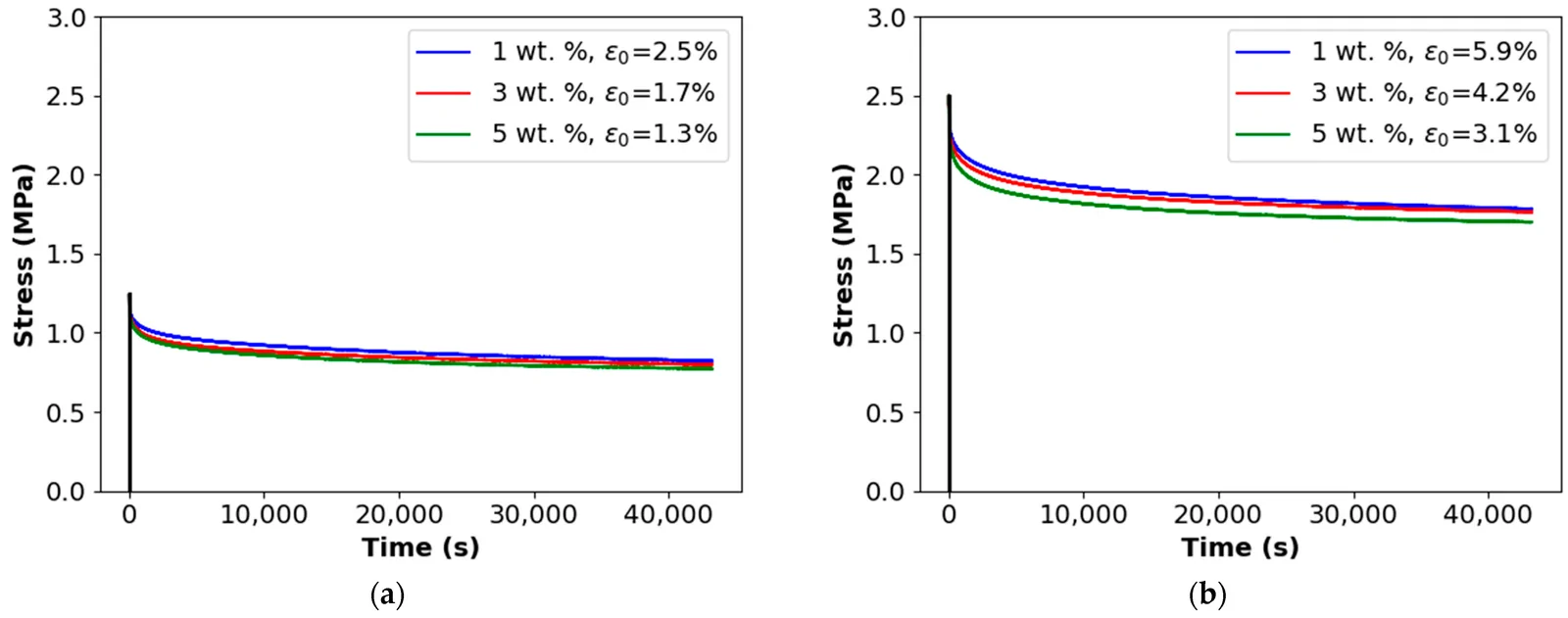
Stress relaxation response of EVA–CNT nanocomposites after stretching to an initial load of (**a**) 50 N and (**b**) 100 N.

**Figure 5:**
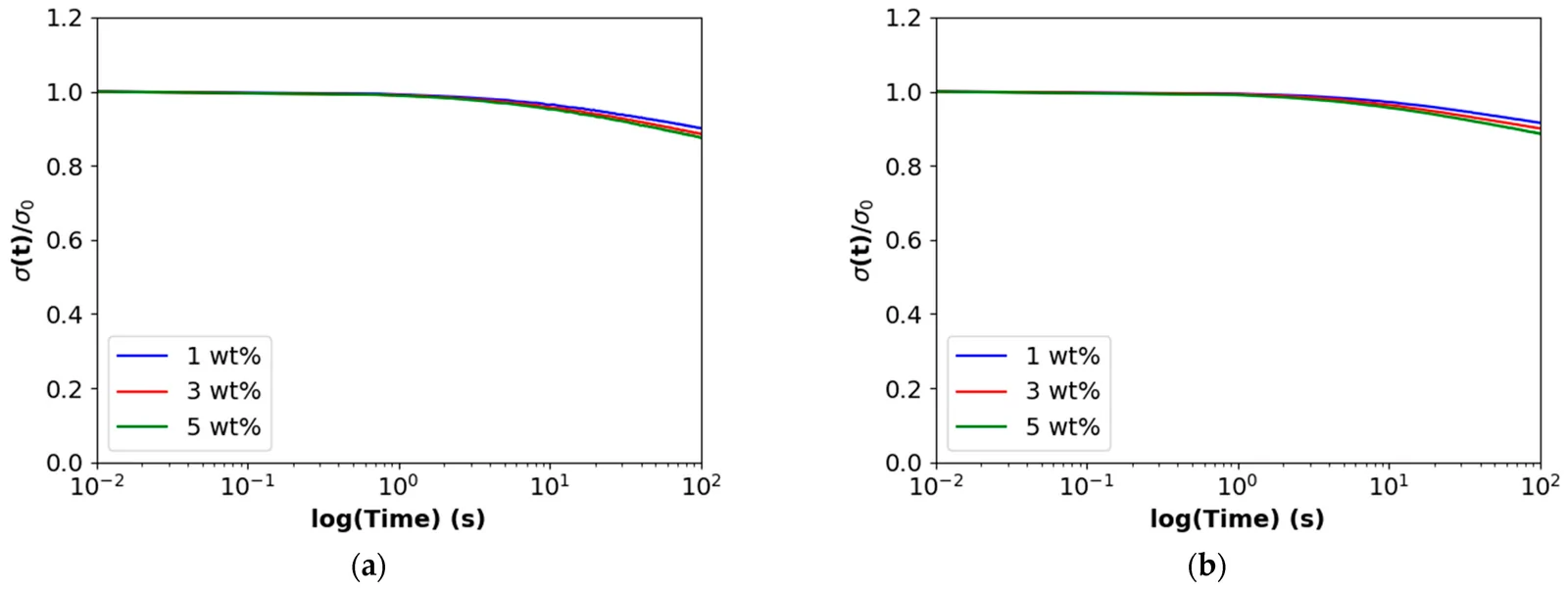
Normalized stress relaxation response of EVA–CNT nanocomposites after stretching 100 s to an initial load of (**a**) 50 N and (**b**) 100 N.

**Figure 6:**
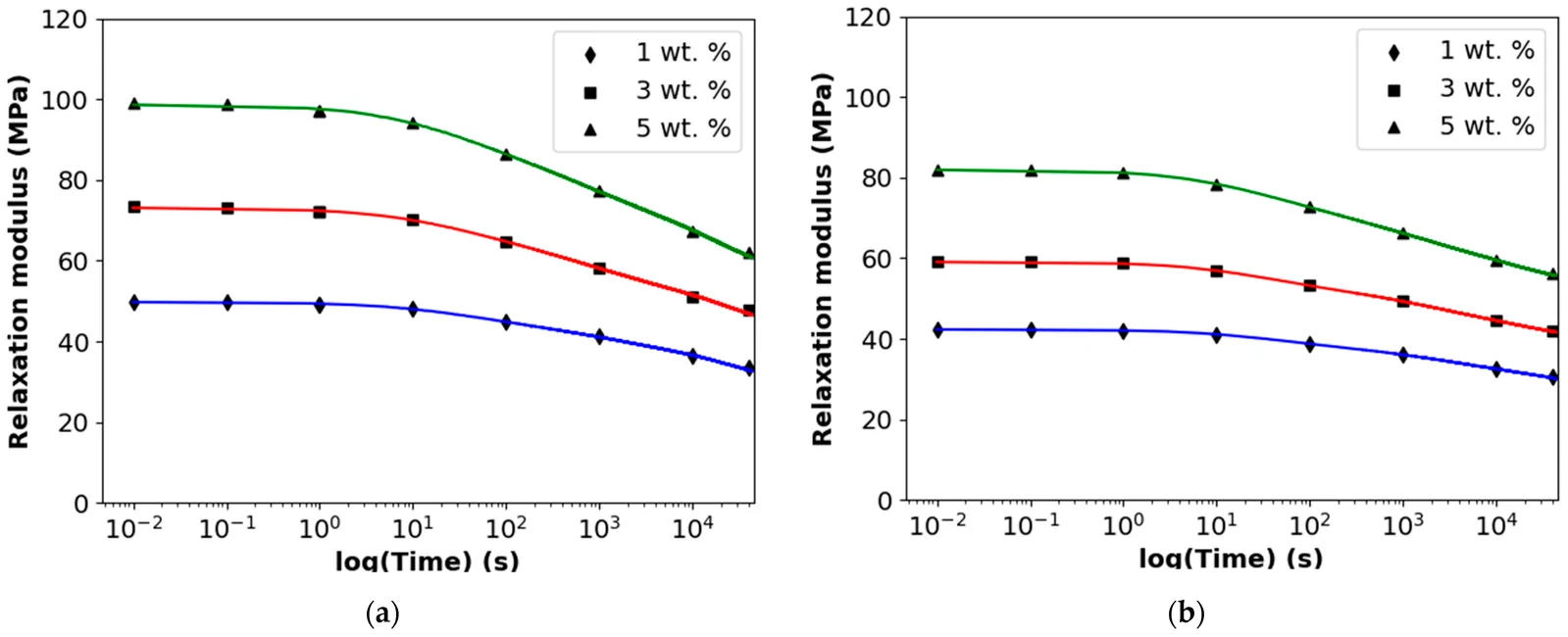
Predicted (symbols) and experimental (solid lines) relaxation modulus of EVA–CNT nanocomposites: (**a**) 50 N and (**b**) 100 N initial loading.

**Figure 7:**
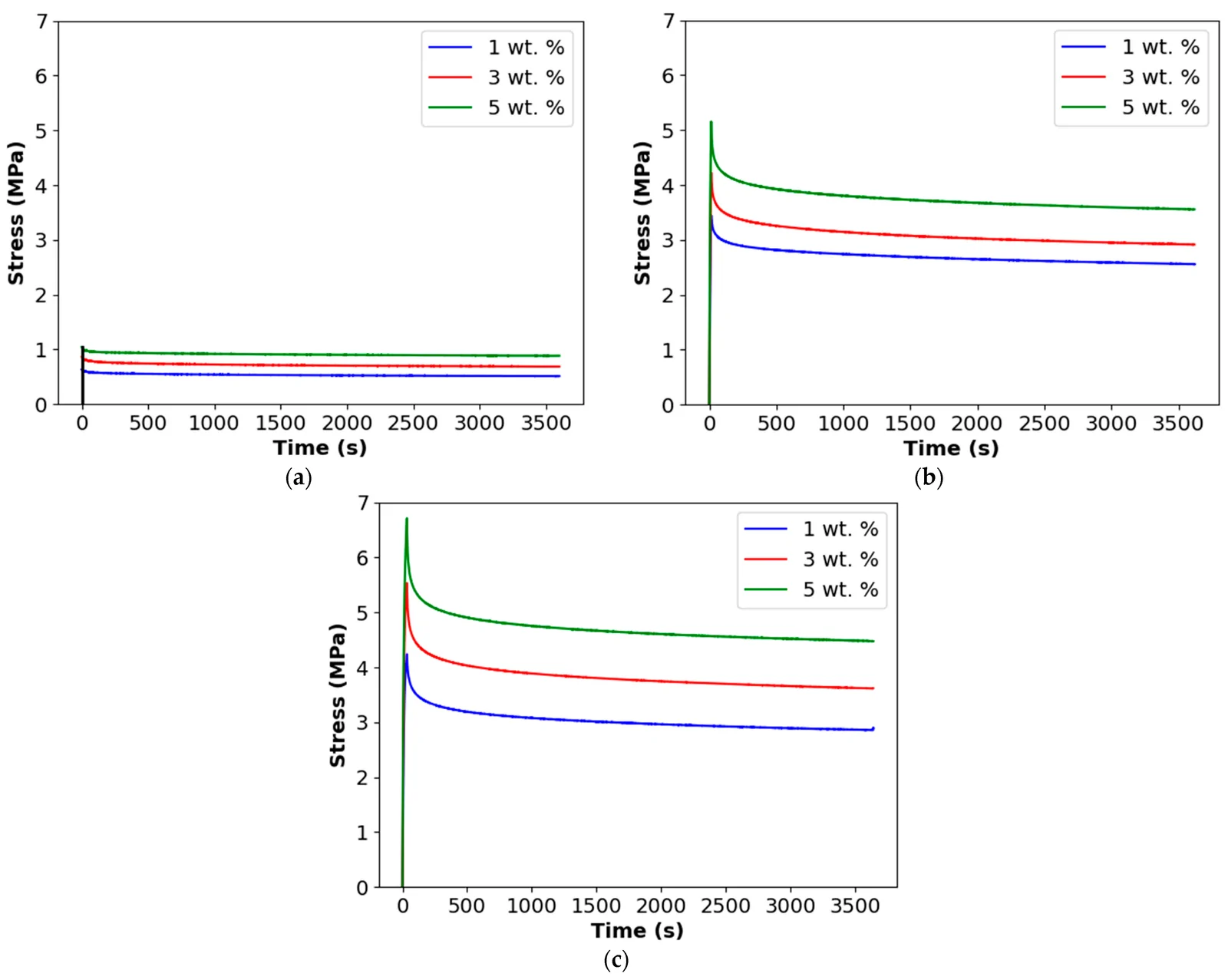
Stress relaxation response of EVA–CNT nanocomposites after stretching to (**a**) 1%, (**b**) 10%, and (**c**) 25% initial strain.

**Figure 8:**
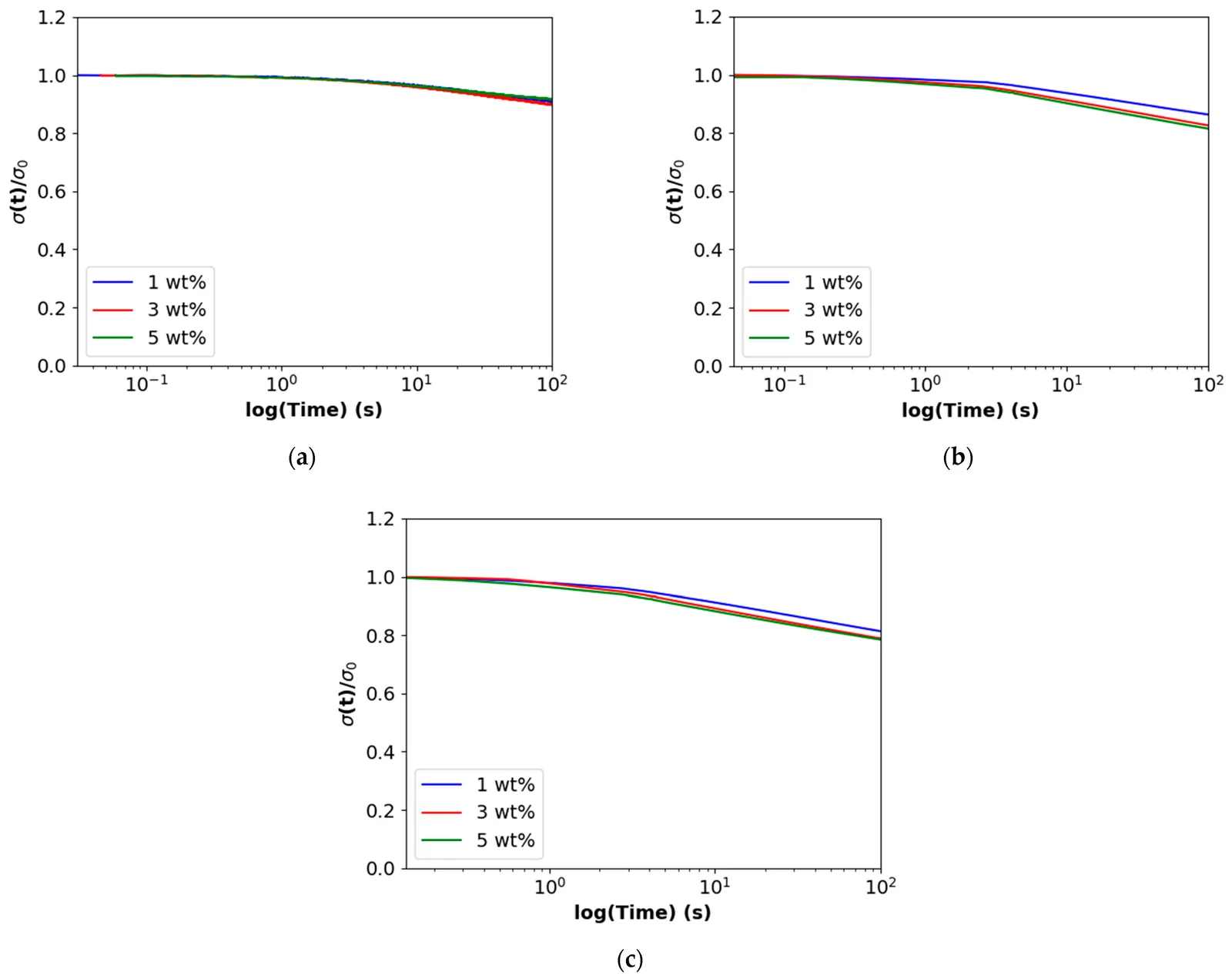
Normalized stress relaxation response of EVA–CNT nanocomposites during the first 100 s relaxation at (**a**) 1%, (**b**) 10%, and (**c**) 25% initial strain.

**Figure 9:**
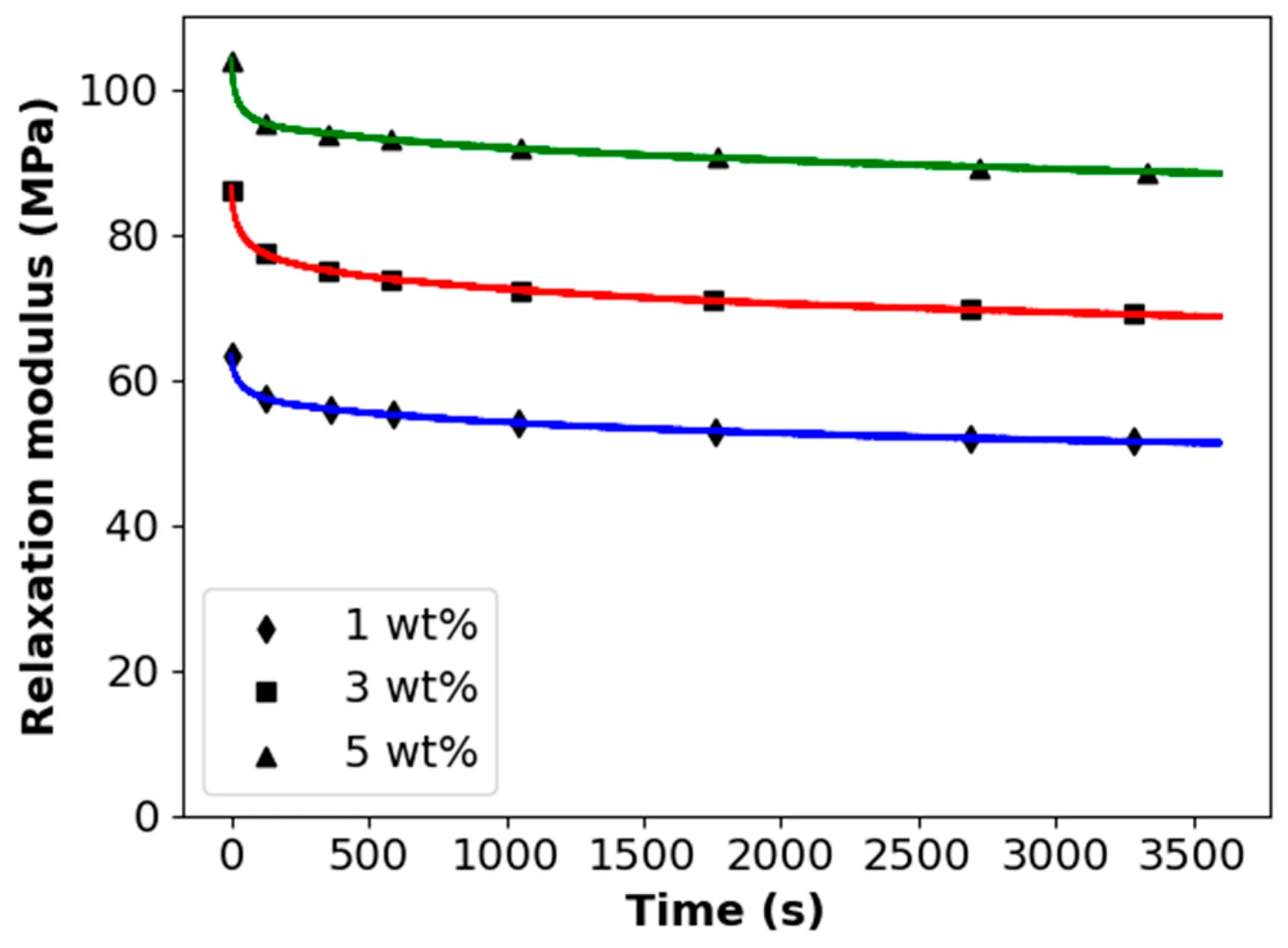
Predicted (symbols) and experimental (solid lines) relaxation modulus of EVA–CNT nanocomposite for 1% strain-controlled test.

**Table 1:** Summary of stress relaxation data for EVA–CNT nanocomposites—load-controlled test.

Initial Load (N)	*σ*_0_ (MPa)	*ε*_0_ (%)	CNTs (wt%)	*σ*_∞_ (MPa)	*R*_∞_ (%)
50	1.25	2.5 ± 0.05	1	0.82 ± 0.0007	34.4
		1.7 ± 0.08	3	0.80 ± 0.0004	36.0
		1.3 ± 0.07	5	0.77 ± 0.0004	38.4
100	2.5	5.9 ± 0.04	1	1.78 ± 0.002	28.8
		4.2 ± 0.11	3	1.76 ± 0.001	29.6
		3.1 ± 0.05	5	1.70 ± 0.001	32.0

**Table 2:** Maxwell parameters of EVA–CNT nanocomposites—the ideal step with 50 N initial loading.

*k*	*τ_k_* (s)	*E_k_* (MPa)		
		1 wt%	3 wt%	5 wt%
1	1	1.15	1.75	2.44
2	10	0.36	1.09	2.04
3	10^2^	5.33	8.39	11.79
4	10^3^	1.90	4.61	6.35
5	10^4^	7.72	9.99	14.54
*E*_∞_ (MPa)		33.47	47.55	61.87
*E*_0_ (MPa)		49.94	73.37	99.03
*R* ^2^		0.982	0.9872	0.991
*SSq*		1.978	1.305	0.225

**Table 3:** Maxwell parameters of EVA–CNT nanocomposites—the ideal step with 100 N initial loading.

*k*	*τ_k_* (s)	*E_k_* (MPa)		
		1 wt%	3 wt%	5 wt%
1	1	8.89 × 10^−2^	1.5 × 10^−2^	0.76
2	10	1.27	2.77	3.30
3	10^2^	3.02	4.13	7.32
4	10^3^	2.14	3.48	5.50
5	10^4^	5.29	6.66	9.03
*E*_∞_ (MPa)		30.48	41.98	56.07
*E*_0_ (MPa)		42.29	59.04	81.98
*R* ^2^		0.994	0.996	0.997
*SSq*		0.639	0.338	0.306

**Table 4:** Summary of stress relaxation data for EVA–CNT nanocomposites—strain-controlled test.

*ε*_0_(%)	CNTs (wt%)	*σ*_0_ (MPa)	*σ*_∞_ (MPa)	*R*_∞_ (%)
1	1	0.63 ± 0.003	0.51 ± 0.018	19.05
	3	0.87 ± 0.009	0.69 ± 0.007	20.69
	5	1.04 ± 0.071	0.88 ± 0.010	15.38
10	1	3.45 ± 0.04	2.56 ± 0.039	25.80
	3	4.23 ± 0.030	2.91 ± 0.081	31.21
	5	5.15 ± 0.044	3.55 ± 0.088	31.07
25	1	4.24 ± 0.034	2.86 ± 0.050	32.55
	3	5.53 ± 0.276	3.61 ± 0.196	34.72
	5	6.71 ± 0.124	4.48 ± 0.003	33.23

**Table 5:** Maxwell relaxation parameters for EVA–CNT nanocomposites: *ε*_0_ = 1%.

*k*	1 wt%		3 wt%		5 wt%	
	*τ_k_* (s)	*E_k_* (MPa)	*τ_k_* (s)	*E_k_* (MPa)	*τ_k_* (s)	*E_k_* (MPa)
1	9.56	2.28	10.71	3.80	7.01	3.00
2	58.84	3.21	71.12	4.74	38.33	4.87
3	513.97	2.30	511.12	3.57	393.49	2.14
4	3217.11	6.22	3599.98	8.34	3599.99	8.54
*E*_∞_ (MPa)	49.20		65.58		85.22	
*E*_0_ (MPa)	63.21		86.03		103.77	
*R* ^2^	0.999		0.999		0.999	
*SSq*	0.041		0.026		0.015	

**Table 6:** Normalized viscoelastic spectra for EVA–CNT nanocomposites: *ε*_0_ = 1%.

Parameter (%)	1 wt%	3 wt%	5 wt%	Time Domain
*g*_1_ (instant)	3.61	4.42	2.89	Fast interfacial slippage (7–10 s)
*g*_2_ (short)	5.08	5.51	4.69	Constrained chain segment motion (38–71 s)
*g*_3_ (medium)	3.64	4.15	2.06	Bulk polymer chain relaxation (393–514 s)
*g*_4_ (long)	9.84	9.69	8.23	Ultimate macromolecular alignment (3200–3600 s)
*g*_∞_ (equilibrium)	77.84	76.23	82.12	Remaining elastic fraction

**Table 7:** Stress relaxation parameters for EVA–CNT nanocomposites: *τ_k_* = 10%.

*k*	1 wt%		3 wt%		5 wt%	
	*τ_k_* (s)	*σ_k_* (MPa)	*τ_k_* (s)	*σ_k_* (MPa)	*τ_k_* (s)	*σ_k_* (MPa)
1	8.17	0.21	7.81	0.35	6.90	0.44
2	51.00	0.20	60.03	0.31	49.64	0.37
3	232.96	0.15	330.23	0.22	275.56	0.29
4	2649.06	0.41	2525.83	0.50	2500.24	0.55
*σ*_∞_ (MPa)	2.45		2.80		3.43	
*σ*_0_ (MPa)	3.42		4.17		5.07	
*R* ^2^	1.000		0.999		1.000	
*SSq*	0.002		0.004		0.002	

**Table 8:** Stress relaxation parameters for EVA–CNT nanocomposites: *ε*_0_ = 25%.

*k*	1 wt%		3 wt%		5 wt%	
	*τ_k_* (s)	*σ_k_* (MPa)	*τ_k_* (s)	*σ_k_* (MPa)	*τ_k_* (s)	*σ_k_* (MPa)
1	7.20	0.34	6.73	0.59	5.97	0.68
2	47.98	0.33	51.39	0.47	44.16	0.54
3	248.52	0.30	293.46	0.38	247.73	0.41
4	2402.20	0.48	2704.13	0.59	2152.91	0.59
*σ*_∞_ (MPa)	2.75		3.46		4.37	
*σ*_0_ (MPa)	4.20		5.49		6.60	
*R* ^2^	1.000		1.000		1.000	
*SSq*	0.003		0.004		0.004	

## Data Availability

The original contributions presented in this study are included in the article. Further inquiries can be directed to the corresponding authors.
